# Treatment of Organic and Sulfate/Sulfide Contaminated Wastewater and Bioelectricity Generation by Sulfate-Reducing Bioreactor Coupling with Sulfide-Oxidizing Fuel Cell

**DOI:** 10.3390/molecules28176197

**Published:** 2023-08-23

**Authors:** Thi Quynh Hoa Kieu, Thi Yen Nguyen, Chi Linh Do

**Affiliations:** 1 Institute of Biotechnology, Vietnam Academy of Science and Technology, 18 Hoang Quoc Viet, Cau Giay, Hanoi 100000, Vietnam; 2 Faculty of Biotechnology, Graduate University of Science and Technology, Vietnam Academy of Science and Technology, 18 Hoang Quoc Viet Str., Cau Giay, Hanoi 100000, Vietnam; 3 Institute of Material Sciences, Vietnam Academy of Science and Technology, 18 Hoang Quoc Viet, Cau Giay, Hanoi 100000, Vietnam

**Keywords:** electricity generation, sulfate-reducing bacteria (SRB), microbial fuel cell, sulfate reduction, sulfide oxidization

## Abstract

A wastewater treatment system has been established based on sulfate-reducing and sulfide—oxidizing processes for treating organic wastewater containing high sulfate/sulfide. The influence of COD/SO_4_^2−^ ratio and hydraulic retention time (HRT) on removal efficiencies of sulfate, COD, sulfide and electricity generation was investigated. The continuous operation of the treatment system was carried out for 63 days with the optimum COD/SO_4_^2−^ ratio and HRT. The result showed that the COD and sulfate removal efficiencies were stable, reaching 94.8 ± 0.6 and 93.0 ± 1.3% during the operation. A power density level of 18.0 ± 1.6 mW/m^2^ was obtained with a sulfide removal efficiency of 93.0 ± 1.2%. However, the sulfide removal efficiency and power density decreased gradually after 45 days. The results from scanning electron microscopy (SEM) with an energy dispersive X-ray (EDX) show that sulfur accumulated on the anode, which could explain the decline in sulfide oxidation and electricity generation. This study provides a promising treatment system to scale up for its actual applications in this type of wastewater.

## 1. Introduction

Organic carbon and sulfate are widespread environmental contaminants resulting from human activities such as tanning processes, chemical manufacturing, landfills, food processing, swine, and the petrochemical industry [1,2,3], Under anaerobic conditions, sulfate-reducing bacteria (SRB) utilize organic compounds as carbon and energy and sulfate as the terminal electron acceptor for sulfide production (H_2_S, HS^−^, S^2−^) and bicarbonate, according to (Equation (1)) below [4,5,6]:

2 CH_2_O + SO_4_^2−^ → H_2_S + 2 HCO_3_^−^ (1)

Therefore, besides organic compounds and sulfate, sulfide is also ubiquitous in these types of wastewater. The organic and sulfide/sulfate contaminated wastewater is a typical corrosive, odorous pollutant and toxic to human health and living organisms, especially in anoxic sulfate-rich environment. This wastewater is a hazardous substance that must be removed from wastewater before discharge into the environment. Sulfide can cause inhibition of the cytochrome oxidase enzyme system resulting in a lack of oxygen use in the cells. Anaerobic metabolism causes the accumulation of lactic acid leading to an acid-base imbalance. The nervous system and cardiac tissues are particularly vulnerable to the disruption of oxidative metabolism and death is often the result of respiratory arrest. Hydrogen sulfide also irritates skin, eyes, mucous membranes, and the respiratory tract. Pulmonary effects may not be apparent for up to 72 h after exposure [7,8,9].

Conventional methods for removing sulfate/sulfide from wastewater include chemical oxidization by chloride (Cl^−^), potassium permanganate (KMnO_4_) and hydrogen peroxide (H_2_O_2_), chemical removal by metal salts [9,10], increasing redox potential to control by sulfide formation by air injection and biological oxidation by sulfide-oxidizing bacteria (SOB) can be used to prevent the formation of sulfide [11]. Although these methods can effectively remove sulfide, they share a common limitation of high energy and chemical consumption, which would result in high operating costs and enormous sludge. Moreover, these methods cannot remove organic carbon and sulfate/sulfide pollutants simultaneously. 

In comparison with chemical methods, sulfide removal by sulfide-oxidizing bacteria (SOB) has the advantages of cost-effectiveness and minimization of chemical sludge [12]. However, SOB is commonly autotrophic so inorganic electron acceptors such as nitrate are needed for sulfide oxidizing. In wastewaters lacking nitrate, simultaneous removal of sulfide is not achievable; while the addition of electron acceptor (nitrate) is not a cost-effective and environmentally friendly option [13,14]. Therefore, it is necessary to seek novel methods to simultaneously remove sulfate/sulfide and organic carbon compounds from wastewater [15].

To solve the problems, microbial fuel cell (MFC), a novel method, which has been considered a promising method in reducing operating costs, energy, and toxic by-products compared with the traditional treatments is proposed in this study [16,17,18]. The use of microbial fuel cells (MFCs) for the treatment of organic carbon wastewater containing sulfate/sulfide attracts great attention nowadays due to the capability of bioelectricity generation and simultaneous removal of sulfate/sulfide and organic compounds based on the dissimilative microbial sulfate-reduction process [15]. Moreover, this technology would generate much less sludge than a conventional activated sludge process [19,20].

In the sulfate-reduction process, sulfate-reducing bacteria were selected as catalysts in wastewater treatment systems. The sulfide produced biologically based on the organic carbon-oxidizing and sulfate-reducing processes by SRB plays a key role of electron mediator in MFCs, which transfers electrons to the anode electrode to produce electricity and the additional amount of synthetic endogenous mediators, toxic and expensive compounds, is not necessary. Then, the released protons in the anodic chamber migrate through a proton—selective membrane into the cathode chamber [21,22]. In the last years, the number of studies on the application of MFC has increased [23].

MFCs have also been employed for effective organic compounds removal with bioelectricity recovery [24,25,26,27,28], while MFCs studies for sulfate/sulfide [2,29,30,31,32], especially simultaneous organics removal which is often presented together with sulfate/sulfide in wastewaters are still limited [33,34].

However, in previous studies, the anodic chamber of MFC was designed to oxidize organic compounds and convert sulfate to sulfide before sulfide oxidation occurs at the anode. This MFC configuration supports the co-existence of sulfate-reducing and sulfide-oxidizing processes in an anodic chamber. However, the main drawback of this configuration is that the MFC trended to have a long hydraulic retention time with a small extent of mixing, which could also have affected the sulfide oxidation and electricity generation of the MFC [31,34]. Therefore, the integration of sulfate-reducing bioreactor and sulfide-oxidizing fuel cell was designed for the improvement of the pollutant removal efficiency in this study.

The aim of this study was to (i) investigate the performance of a wastewater treatment system in the treatment of organic wastewater containing high sulfate under continuous operation and (ii) evaluate the influence of COD/SO_4_^2−^ ratios and hydraulic retention time (HRTs) on the performance of the treatment system. The success of this study will help minimize environmental pollution and human health protection.

## 2. Results

### 2.1. Effect of Different Ratios of COD/SO_4_^2−^ on Removal Efficiencies of Sulfate and COD in SRRB

Operating factors could affect the performance of sulfate-reducing bioreactor (SRRB) and sulfide-oxidizing fuel cell (SOFC) thus typical factors such as COD/SO_4_^2−^ ratio and HRT were investigated. Nine different initial ratios of COD/SO_4_^2−^ (0.5, 1, 1.5, 2, 2.5, 3, 4, 5, and 6) were conducted with sulfate concentration of 1300 mgL^−1^ and HRT fixed at 72 h in SRBR. It was found that both sulfate and COD removals seemed negatively correlated with the initial COD/SO_4_^2−^ ratio, due to its inhibition to SRB.

#### 2.1.1. Effect of Different COD/SO_4_^2−^ Ratios on Sulfate Removal Efficiency in SRBR

**Figure 1:**
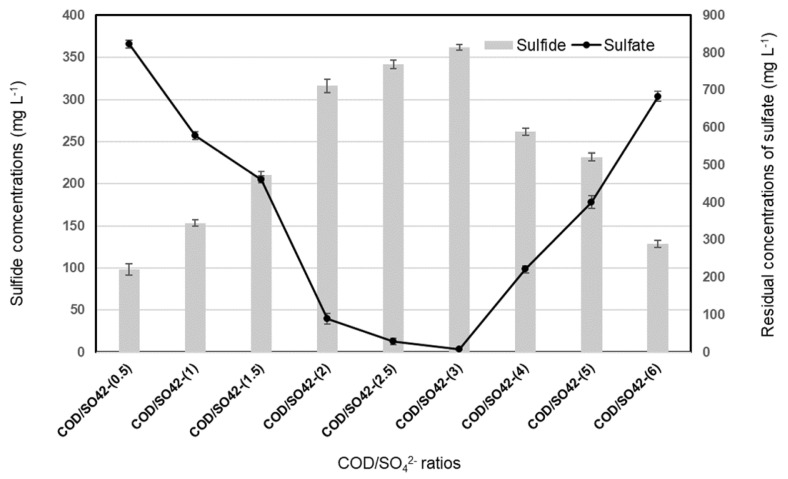
Effect of different ratios of COD/SO_4_^2−^ on sulfate reduction and -sulfide production in SRBR with time. Error bars denote standard deviations.

The COD/SO_4_^2−^ ratio was an important parameter related to electron flow in anaerobic fermentation. Results (Figure 1) indicated that when the ratios of COD/SO_4_^2−^ increased, sulfide production was improved. The lowest sulfide production of 99 ± 7 mg L^−1^ with a sulfate reduction efficiency of 37.1% was obtained with a COD/SO_4_^2−^ ratio of 0.5. When the ratios were increased to 1, 1.5, 2, 2.5, and 3, the sulfide production improved up to 153 ± 4, 213 ± 4, 320 ± 8, 344 ± 5, 364 ± 3 mg S L^−1^ with sulfate reduction of 55.8, 65, 93.5, 98.2 and 99.5%, respectively. The strong sulfate-reducing activity was observed at a COD/SO_4_^2−^ ratio of 3 with a maximum of sulfide production (364 ± 3 mg L^−1^) and sulfate reduction (99.5%). The results showed that at COD/SO_4_^2−^ ratios higher than 3, sulfate removal efficiency decreased gradually. Sulfate was converted by 83.5, 69.4, and 47.8% of initial concentrations to sulfide of 263 ± 4, 233 ± 5, and 128 ± 4 mg L^−1^ under the feed COD/SO_4_^2−^ ratios of 4, 5, and 6, respectively.

#### 2.1.2. Effect of Different COD/SO_4_^2−^ Ratios on COD Removal Efficiency in SRBR

**Figure 2:**
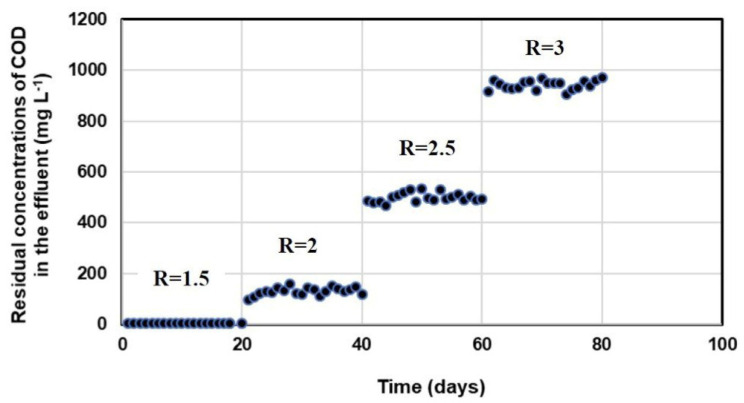
Residual concentrations of COD at various COD/SO_4_^2−^ ratios in SRBR with time (R = COD/SO_4_^2−^ ratio).

Figure 2 shows the residual concentrations of COD at various COD/SO_4_^2−^ ratios (1.5; 2; 2.5, and 3). These results indicate that when the COD/SO_4_^2−^ ratio increased to more than 1.5, sulfate reduction and sulfide production were improved (Figure 1), whereas COD was incompletely oxidized (Figure 2).

At the COD/SO_4_^2−^ ratio of ≤1.5, the COD removal efficiency was 99.7 ± 0.3%, and COD was not detected in the effluent. When the initial COD/SO_4_^2−^ ratio became 2 by adjusting the lactate concentration in the influent, the COD removal efficiency decreased to 95 ± 0.6%. At an initial COD/SO_4_^2−^ ratio of 2.5 and 3, the COD removal efficiency dropped further to 84.6 ± 0.5 and 75.8 ± 0.5%. COD removal was close to 100% at feed COD/SO_4_^2−^ ratios between 0.5 and 1.5. At higher ratios (2; 2.5 and 3), COD was detected in the effluent with residual COD concentration of 130 ± 15.6; 499 ± 17.7; and 942 ± 18 mg L^−1^, respectively (Figure 2). This can be attributed to the sulfate-limiting conditions (Figure 1). The incomplete oxidation of COD was compensated by adding an excess of lactate to obtain the high sulfide production at the feed COD/SO_4_^2−^ ratio of 1.5, 2, 2.5, and 3, with sulfate removal efficiencies of 65, 93.5, 98.2, and 99.5%, respectively (Figure 1). Based on the efficiencies of sulfate and COD removal, the COD/SO_4_^2−^ ratio of 2 was selected for further determination of a suitable HRT in SOFC.

### 2.2. Effect of HRTs on Sulfide Removal and Electricity Generation in SOFC

Different levels of HRT (12, 18, and 24 h) were examined with the average initial sulfide concentrations of 316 ± 5.8 mg L^−1^. The power density was calculated by voltage and current every 2 h in SOFC during operation. With prolonged HRT, the removal of sulfide was enhanced. Microbes could have sufficient opportunity to contact and react with sulfide when HRT increased.

**Figure 3:**
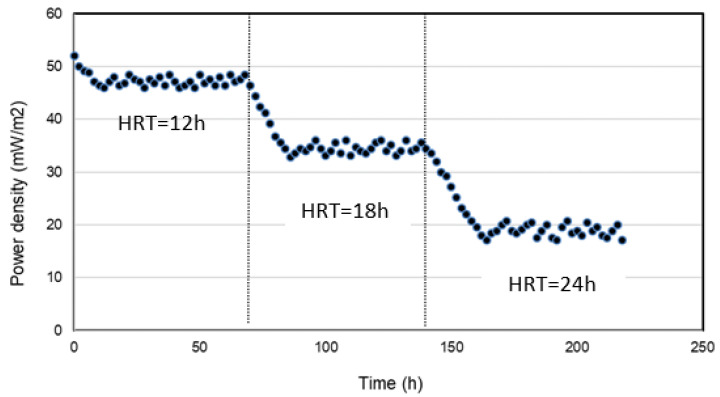
Power density in the SOFC at different HRTs (12, 18 and 24 h) with time.

**Figure 4:**
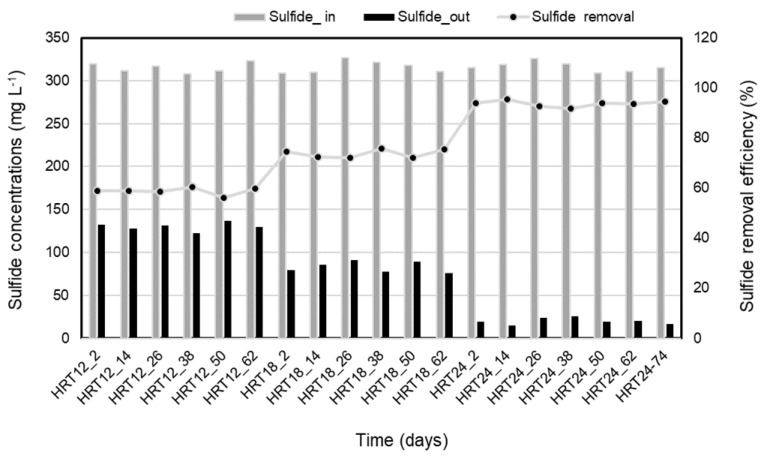
Sulfide removal efficiency in the SOFC at different HRTs (12, 18 and 24 h) with time.

Figure 3 and Figure 4 show the results of power density and sulfide removal in SOFC. At an HRT of 12 h, the highest power density was observed, reaching 47.1 ± 0.9 mW/m^2^. Meanwhile, the sulfide concentration in the effluent was 130 ± 4.9 mg L^−1^, corresponding to sulfide removal efficiency of 58.8 ± 1.5%. As the HRT increased, the sulfide removal efficiency increased, while power density dropped. At the HRT of 18 and 24 h, the power densities of 34.5 ± 1 and 18.9 ± 1.1 mW/m^2^ were obtained, respectively, with the stabilized voltage was 0.02 V. The sulfide concentrations of HRT 18 and 24 h in the effluent of SOFC were 83 ± 6.3 and 20 ± 3.8 mg L^−1^, corresponding to sulfide removal efficiencies of 73.7 ± 1.7 and 93.7 ± 1.2%, respectively (Figure 4). The maximum sulfide removal efficiency achieved was as high as 93.7 ± 1.2% with HRT of 24 h.

### 2.3. The Removal of COD, Sulfate, Sulfide and Electricity Generation in Wastewater Treatment System

Based on the previous experiments, continuous operation of the wastewater treatment system (SRBR integrated with SOFC) was set up with the optimum COD/SO_4_^2−^ ratio of 2 and sulfate concentration of 1300 mg L^−1^. The experiment was carried out for 63 days. The synthetic wastewater (see Section 4.2.1) was fed continuously at the bottom of the SRBR by a peristaltic pump with HRT of 72 h. The effluent of SRBR was supplied using a peristaltic pump into the anode of SOFC at HRT of 24 h.

**Figure 5:**
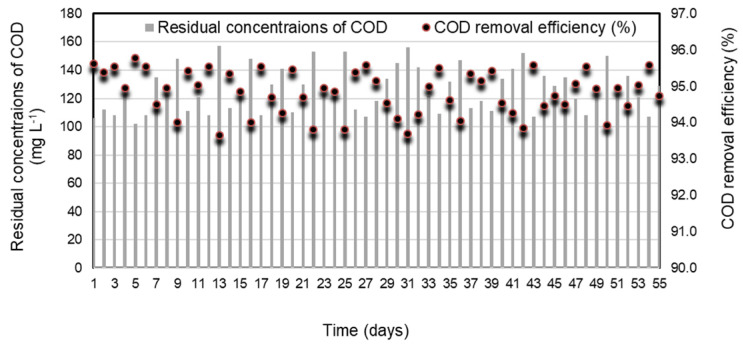
Removal efficiency and residual concentrations of COD in SRBR with time.

**Figure 6:**
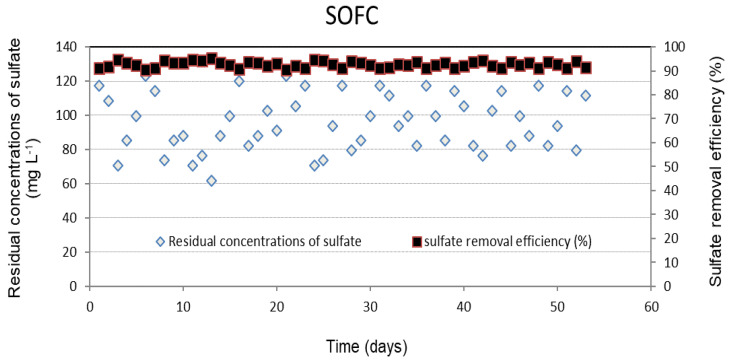
Removal efficiency and residual concentrations of sulfate in SRBR with time.

* Removal efficiency of COD and sulfate in the SRBR:

Figure 5 and Figure 6 display the daily performance outcomes of the SRBR including the removal efficiencies and residual concentrations of COD and sulfate under the selected conditions. The obtained results showed that the concentrations of COD and sulfate decreased significantly after 7 days. The lag period observed in the initial phase was attributable to the slow initiation of activities of SRB. The COD and sulfate removal efficiencies were stable during the continuous operation from day 8 to 63, with an average COD removal efficiency of 94.8 ± 0.6% (Figure 5). Sulfate was converted by 93 ± 1.3% of the initial concentration to sulfide (Figure 6).

* Sulfide removal and electricity generation in SOFC

**Figure 7:**
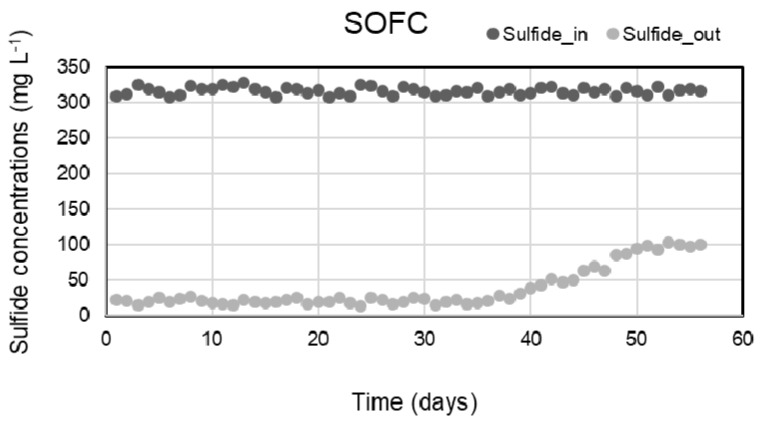
Sulfide concentrations in the influent and effluent of SOFC with time.

In this study, the anode chamber was filled with the effluent of the SRBR at HRT of 24 h during 63-day operation with the average sulfide concentrations of 316 ± 5.8 mg L^−1^. Concentrations of sulfide in influent and effluent of the SOFC are shown in Figure 7. Within 38-day operation from day 8 to 45, sulfide was removed stably from the anode chamber with an amount of 93 ± 1.2%. The average sulfide concentration in the effluent of SOFC was 21 ± 3.8 mg L^−1^. This means that the sulfide present in the anode solution was oxidized, releasing electrons to the anode when the SOFC operation began, resulting in electricity production.

**Figure 8:**
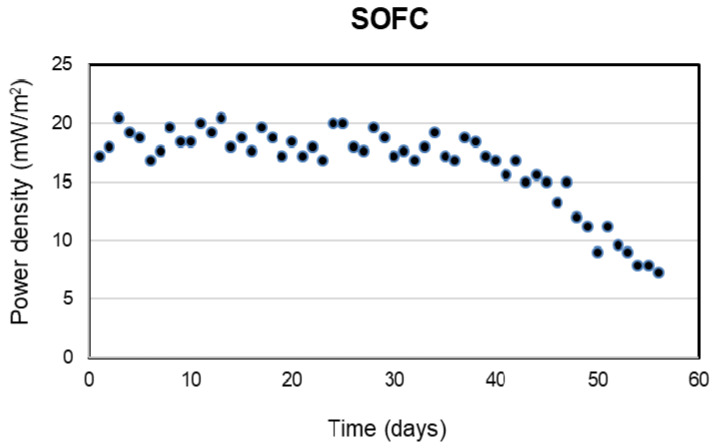
Power density during continuous operation of SOFC with time.

Electricity was generated continuously from the sulfide oxidation process of SOFC during 63-day operation. The power density reached a maximum power density (18.2 ± 1.6 mW/m^2^) after the first week and remained stable for the next 35 days of SOFC operation (days 8 to 43). During the early stage of the SOFC operation, sulfide oxidation in the SOFC appeared to correspond to electricity generation. Figure 7 and Figure 8 showed the relative positive correlation between bioelectricity generation and initial sulfide concentration, due to the reason that the anode potential decreased with the increase in sulfide as it possesses lower redox potential.

However, from day 43 to 45 onwards, the sulfide removal efficiency and power density dropped gradually to 68% and 7.2 mW/m^2^, respectively, at the end of the operation (on day-63) (Figure 7 and Figure 8).

**Figure 9:**
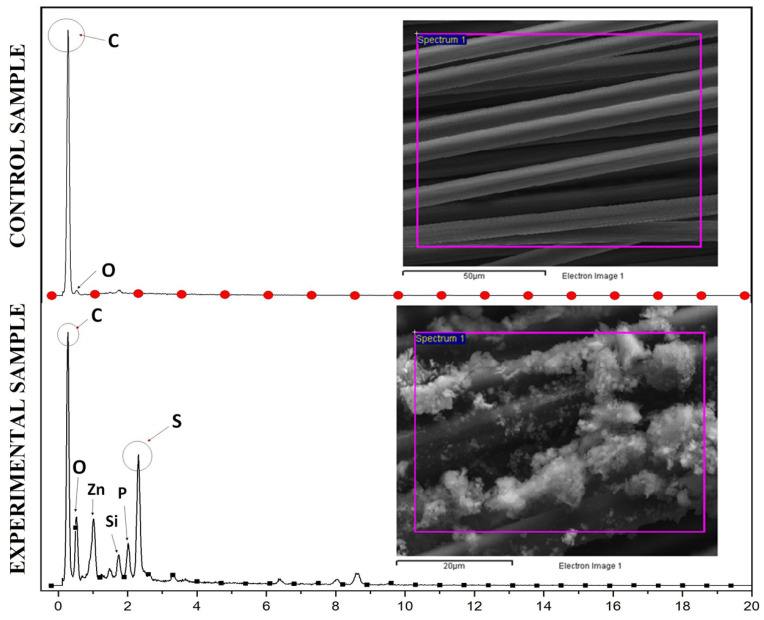
SEM images and EDX analysis of the anode surface before (**Above**) and after (**Below**) SOFC operation.

The results of anode surface analysis by SEM-EDX are shown in Figure 9. As presented in Figure 9, many solid deposits were formed on the anode surface after SOFC operation. The characterization of the solids using EDX revealed that their major component was elemental sulfur. These indicate that sulfide was oxidized at the anode surface, resulting in the formation of insoluble elemental sulfur.

## 3. Discussion

The performance of the wastewater treatment system in this study revealed that a SOFC can be integrated effectively with a SRBR. To minimize environmental pollution, the SOFC was operated without the addition of synthetic electron transport mediators during the operation. The produced sulfide from the sulfate-reducing process in SRBR, a biological compound, was used as an electron mediator, which transfers electrons to the anode electrode [35,36]. Sulfide can be removed in the SOFC when it is oxidized through electrochemical reactions at the anode. Electrons and protons were produced during the process of oxidation of sulfide. The resulting electrons are delivered to the anode and transported to the cathode through an external circuit, producing electricity. Then, the released protons in the anodic chamber migrate through a proton exchange membrane (PEM) into the cathode chamber [36,37].

Oxygen, ferricyanide, nitrate, persulfate and permanganate are widely used as electron acceptors in the cathode due to their high oxidation potential in the cathode. In this study, the protons are taken up and consumed by ferricyanide and oxygen. Both ferricyanide and oxygen in the presence of electrons donated from the cathode surface react with protons and are reduced to ferrocyanide and water, as shown in Equations (2) and (3) [2,38,39]:

O_2_ + 4e^−^ + 4H^+^ → 2H_2_O (2)

Fe (CN)_6_^3+^ + e^−^ → Fe (CN)_6_^4+^ (3)

Using a sulfide mediator to produce electricity was observed in different studies. Ref. [35] reported that dissolved sulfide can be converted to elemental sulfur by MFCs. The MFCs were connected with an up-flow anaerobic sludge reactor, providing removal of up to 98% and 46% of the sulfide and acetate, respectively. Ref. [40] studied the electricity generation potential and demonstrated the anodic potential was controlled by the sulfide concentrations in the chamber when treating sulfate-laden wastewaters. These investigations demonstrated that simultaneous sulfide and organic carbon removals with electricity generation can be achieved in MFCs. Ref. [29] studied sulfide removal by MFCs prior to irrigation water reuse. Ref. [33] demonstrated that removal efficiencies of 49.7 and 70% of the initial sulfide concentrations (150 and 60 mg L^−1^) and power density of 1.2 mW/m^2^ were achieved in the continuous MFC.

The ratio of COD/SO_4_^2−^ and HRT influences sulfate-reducing and COD oxidizing capability of SRB [41,42,43]. In this study, they were recognized as the most two important factors for sulfate and COD removal in SRBR as well as sulfide removal and bioelectricity generation in SOFC. The obtained results showed that at initial COD/SO_4_^2−^ ratios of 2–3 most of the sulfate in the influent was converted to sulfide with sulfate reduction of 93.5–99.5%. The finding was similar to results obtained by Ref. [44], who reported sulfate removal efficiencies over 91% at COD/SO_4_^2−^ ratios equal to or higher than 2.5 for sulfate concentrations up to 1960 mg L^−1^**.**

On the other hand, the obtained data (Figure 1) showed that the lower (0.5, 1, 1.5) or higher COD/SO_4_^2−^ ratios (4, 5, 6) might result in low sulfate removal efficiency in the SRBR (37.1 to 83.5%). The lowest sulfide production with sulfate reduction efficiency of 37.1 and 47.8% were observed with COD/SO_4_^2−^ ratios of 0.5 and 6, respectively. COD removal was close to 100% at COD/SO_4_^2−^ ratios ≤ 1.5. At higher ratios (≥2), COD was detected in the influence of the SRBR. Low sulfate reduction at COD/SO_4_^2−^ ratios of 0.5, 1, and 1.5 might be attributed to the inhibition of the anaerobic process due to the lack of carbon source [45]. However, at high COD/SO_4_^2−^ ratios (4, 5, and 6) and in sulfate-limiting conditions, methane-producing bacteria (MPB) might be dominant in the competition with SRB. Ref. [46] demonstrated that the highest sulfate removal efficiency was obtained when lactate or acetate was used as carbon and electron sources at COD/SO_4_^2−^ ratios between 1.5 and 2.25. Ref. [47] also suggested that a negative effect on the SRB activity can be observed at COD/SO_4_^2−^ ratios of more than 2.7 because competition for nutrients can occur between SRB and MPB. Ref. [43] reported that COD/sulfate ratio and HRT influence sulfate loadings and were recognized as the most two important factors for sulfate removal and bioelectricity generation. In their study, the maximum electricity generation and sulfate removal (83.9%), with the fixed COD/sulfate ratio of 4 and the influent COD of 2400 mg L^−1^, were established at an HRT of 60 h. However, as mentioned above, COD was detected in the influence of the SRBR at COD/SO_4_^2−^ ratio equal to or higher than 2. Therefore, based on the efficiencies of sulfate and COD removal, the COD/SO_4_^2−^ of 2 was selected for the 63-day -continuous operation.

Besides the COD/SO_4_^2−^ ratio, HRT is also one of the most important factors during the operation of biological processes, as it determines the contact duration between pollutants and microbes. In this study, the maximum sulfide removal efficiency achieved was as high as 93.7 ± 1.2% with HRT of 24 h. However, the highest power density was observed, reaching 47.1 ± 0.9 mW/m^2^ at HRT of 12 h. This might explain that the higher sulfide removal efficiency at the increased HRT resulted from the slower flow with the longer retention time providing sulfide ions more opportunities to undergo electrochemical reactions on the anode. In terms of the power density performance, however, a slower flow for a higher HRT is undesirable due to the lack of the supply of fresh sulfide ions from SRBR. In such cases, the anode loses the opportunity to encounter electron-rich fresh sulfides, thereby undergoing mass transport polarization. Electricity generation displayed an inverse phenomenon as electron donors (sulfide) became insufficient. The abundant consumption of sulfide also raised the anode potentials which did not benefit bioelectricity generation.

MFCs often faced trade-off in aspects of electricity generation and pollutant removal [48]. Based on the consent MFC technology should be applied as a waste/wastewater treatment unit rather than a renewable energy source. In this study, considering the sulfide removal efficiency, which is one of the most important values in a wastewater treatment system, the optimum HRT was determined to be 24 h. The continuous operation of the treatment system was carried out for 63 days with the optimum COD/SO_4_^2−^ ratio and HRT. The results indicated that the COD and sulfate removal efficiencies were stable in SRRB, reaching 94.8 ± 0.6% and 93 ± 1.3% during the operation. Electricity was generated continuously and stably (18 ± 1.6 mW/m^2^) from the sulfide oxidation process of SOFC for the first 45 days with 93 ± 1.2% of the sulfide being oxidized from the anode chamber. However, the sulfide removal efficiency and power density decreased gradually after 45 days. At the end of the operation, sulfide removal efficiency and power density were 68% and 7.2 W/m^2^, respectively.

The results obtained in our study showed that the generated bioelectricity is proportional to the sulfide concentration, the higher concentration of sulfide obtained, the more electricity is generated. The results are consistent with the report from [40,49]. They demonstrated that the bioelectricity produced by the electrodes was dependent on the concentration of the sulfide, which on the other hand indicated the sulfide oxidation process.

The decline in sulfide oxidation and electricity generation after 45 days of operation can be attributed to the deposition of elemental sulfur, which hinders the effective mass transport of fresh sulfide ions to the anode electrode. The concentration of sulfate did not increase during the sulfide removal, suggesting that under this condition, sulfide was oxidized to sulfur, not sulfate. This was confirmed through an anode surface analysis with SEM and EDX at the end of the SOFC operation. The accumulation of sulfur on the anode may have decreased the electrical conductivity of the anode, thereby increasing the overpotential of the anode in the SOFC over time. This explanation is in agreement with previous studies [50,51]. Ref. [50] observed losses in the current output of MFCs and the reduction in removal of sulfide or sulfate from wastewater due to the deposition of elemental sulfur on the electrode surface. Furthermore, exopolysaccharides produced from SRB hamper electron transfer between bacterial cells and the electrode and thus reduce the voltage. Ref. [52] who investigated the performance of MFC treating organic wastewater containing high sulfate showed that the ohmic loss or internal resistance of the MFC increased over time from day 13 to day 54. The anode replacement on day 81 resulted in a significant reduction in the ohmic loss, suggesting the important role of the anode in the internal resistance of the MFC.

To remove effectively pollutants from wastewater and generate stable electricity in long-term operation, the integrated treatment system should be (i) separated from sulfate-reducing and sulfide-oxidizing processes, and (ii) operated in continuous flow mode. In such systems, substrate and other nutrients will be continuously supplied to the SRB. Moreover, the continuous flow mode could also remove the by-product elemental sulfur from the anode compared with MFCs operated in batch mode. Furthermore, the presence of sulfate in the anode media has negative effects on sulfide oxidation and electricity generation in MFCs. Ref. [31] reported that low concentrations of sulfate (≤1470 mg L^−1^) benefited the MFC efficiency, while higher sulfate presence blocked the sulfide oxidization and electricity generation.

## 4. Materials and Methods

### 4.1. Inoculum and Culture Medium

A consortium of SRB was enriched from anaerobic sludge rich in sulfide from a crude oil tanker, Vung Tau, Vietnam, and used as the inoculum. This culture was cultivated under anaerobic conditions using Postgate’s medium B [53] with a slight modification. Modified Postgate’s B medium contained (in g/L): KH_2_PO_4_ 0.5; NH_4_Cl 1.0; Na_2_SO_4_ 1.0; MgSO_4_·7H_2_O 2.0; Sodium lactate 3.2; Yeast extract 0.5; FeSO_4_.7H_2_O 0.01; ascorbic acid 0.1; thioglycolic acid 0.1. The pH was adjusted to 7.0 ± 0.2 using HCl 1 M or NaOH 1 M.

All procedures during the preparation of the medium and cultivation were performed according to the modified Hungate’s method for anaerobes [54]. To enrich the SRB number, the cultivation step was repeated three times before inoculating into the SRBR. The enrichment culture was obtained as follows: The culture was seeded with 10% (*v*/*v*) inoculum and incubated at 30 °C in a Hungate tube or glass culture bottle. The medium was sparged with pure nitrogen gas to maintain anaerobic conditions before inoculation. Every week 10% of the volume of the culture in the bottle (or Hungate tube) was replaced by fresh medium. After 3-time cultivations, a culture containing a high density of SRB was achieved. The cell density of the enrichment culture was approximately 1 × 10^8^ cells mL^−1^ in all experiments.

### 4.2. Design of Wastewater Treatment System

**Figure 10:**
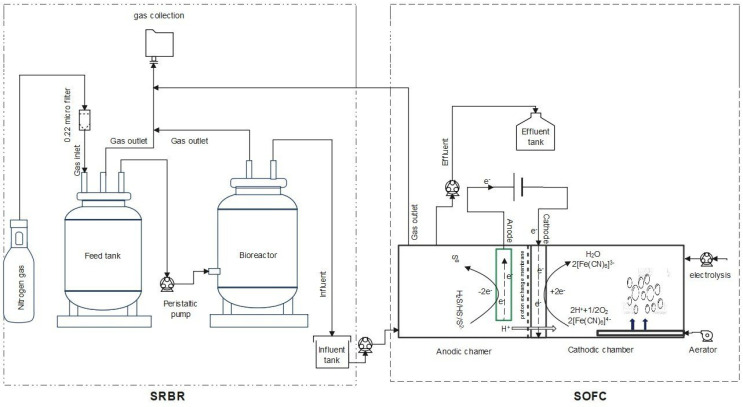
Configuration of a sulfate-reducing bioreactor (SRBR) integrated with a sulfide-oxidizing fuel cell (SOFC).

The system consisted of two identical components: (1) a SRBR to reduce sulfate to sulfide; (2) a SOFC to oxidize subsequently sulfide to element sulfur (S°)/sulfate (SO_4_^2−^). The integrated use of SRBR and SOFC can remove organic matter (COD), sulfate and sulfide simultaneously with electricity generation. The experimental apparatus is illustrated in Figure 10.

#### 4.2.1. Sulfate-Reducing Bioreactor (SRBR)

The schematic diagram of the feeding tank and sulfate-reducing bioreactor are present in Figure 10.

* Feeding tank

Synthetic wastewater (see below) was prepared aseptically to avoid contamination and then fed continuously at the bottom of the bioreactors by a peristaltic pump (Ismatec SA, Zuerich, Switzerland) with a volumetric flow of 125 mL d^−1^ (HRT of 72 h).

To maintain the anaerobic condition, feeding tank was purged with filter sterilized nitrogen gas (0.22 µm). Gas produced during the treatment process was trapped by 4% (*w*/*v*) NaOH solution.

* Synthetic wastewater composition

The composition of the synthetic wastewater (g L^−1^) consisted of KH_2_PO_4_, 0.5; NH_4_Cl; Na_2_SO_4,_ 1.9; MgSO_4_·7H_2_O, 0.06. Synthetic wastewater was fed with different COD/SO_4_^2−^ ratios. The sulfate concentration (1300 mg L^−1^) was maintained constant during the whole experiment and the sodium lactate concentration (in COD) was varied in the medium (1070, 2140, 3210, 4280, 5350, 6420, 8560, 10,700 and 12,840 mg L^−1^) to obtained feed COD/SO_4_^2−^ ratios of 0.5, 1.0, 1.5, 2.0, 2.5, 3, 4, 5 and 6, respectively. The synthetic wastewater was not supplemented with Fe^2+^ and reducing agents such as yeast extract, ascorbic acid, sodium thioglycolate and Na_2_S to prevent the precipitation of FeS. This avoids clogging pipes and membranes, and loss of sulfide in the treatment system. The pH was adjusted to 7.0 ± 0.2 using HCl 1 M or NaOH 1 M.

* SRBR and operating conditions

For sulfate reduction and removal of organic matter (COD), the experiments were carried out in a continuous anaerobic SRBR in an up-flow mode. This reactor was fabricated from glass, having a total volume of 350 mL and a working volume of 250 mL. The SRBR was soaked in a 3 M HNO_3_ solution for 72 h and rinsed with de-ionized water before use to avoid contamination.

The SRBR was first inoculated with 10% (*v*/*v*) of the enriched SRB consortium containing 1 × 10^8^ cells mL^−1^ using synthetic wastewater containing sulfate and sodium lactate as electron acceptor and donor, respectively. The synthetic wastewater was continuously injected into the bottom of the SRBR by a peristaltic pump. It then flowed upward. After inoculation with enriched SRB consortium, the reactor was purged by N_2_ gas to provide the anaerobic condition. To investigate the roles of SRB in the removal of organic (COD) and sulfate in SRBR, the reactor was operated continuously with a HRT of 72 h for 63 days at ambient room temperature (25 ± 2 °C).

#### 4.2.2. Sulfide-Oxidizing Fuel Cell (SOFC)

* Design and fabrication of SOFC

Double-chambered SOFC design was used to investigate the removal of sulfide and electricity generation in SOFC. The SOFC consisted of two identical chambers (working volume of 80 mL each): (i) anode chamber, where electrochemical oxidation of sulfide on an anode surface derives electrons and protons and (ii) cathode chamber, where the oxygen and ferricyanide are the terminal electrons acceptors and react with the released protons. The design of this SOFC precludes the possibility of oxygen ingress in the anodic chamber because the anode and cathode chambers are divided by proton-exchange membrane (PEM). It is permeable only for H^+^ cations.

Both chambers were made of mica acrylic plates to prevent any corrosion from sulfide. These plates were fabricated by using a CNC machine. After machining, the plates were glued with acrylic resin to form the SOFC chambers. Before the experiment, all the electrodes were first immersed in 1 M NaOH then 1 M HCl for one-hour each to remove microbial residues on the electrodes surface.

* Preparation of SOFC electrode

An electrocatalyst ink was prepared by mixing Pt/C 40% wt. catalyst powder (Johnson Mathey—Wayne, PA, USA), Nafion solution 5% and iso-propanol. Afterward, the mixture was ultrasonicated and stirred by magnetic to ensure that the catalyst powder was uniformly dispersed in the ink. The Pt/C catalyst layer was prepared by brushing the catalyst ink on a carbon cloth with an active area of around 10 cm^2^. After each brushing, the sample was dried in air and this process was repeated until reaching the desired Pt loading of 1 mg/cm^2^. Finally, the catalyst layer was dried at 130 °C for 30 min. The cathodic electrode was fabricated by hot pressing the catalyst layer on a Nafion 117 membrane. The hot-pressing conditions were executed at a temperature of 135 °C, duration time of 180 s and pressure of 21 kg/cm^2^. For the anode, a plain carbon cloth (1071 HBC, Pinon Driver,—USA) was utilized with a working area of about 5 cm^2^.

* SOFC operation conditions

To investigate the feasibility of sulfide removal and the electricity generation capacity of the SOFC, the effluent of SRBR was fed continuously into the anode chamber of SOFC by a peristaltic pump with HRT of 24 h. The SOFC was operated for 63 days at ambient room temperature (25 ± 2 °C).

The cathode chamber was filled with ferricyanide solution (50 mM K_3_Fe (CN)_6_) and air was supplied through a diffuser connected to an aerator. Ferricyanide solution is often used in the cathodic medium in SOFC to substitute oxygen as a cathodic electron acceptor due to low overpotential. To maintain sufficient deoxidation by oxygen in the cathode chamber, fresh ferricyanide solution was replenished in the cathodic chamber every week.

#### 4.2.3. Determination of Suitable Conditions for SRBR and SOFC

* Determination of COD/SO_4_^2−^ ratios for sulfate removal in SRBR

To select a COD/SO_4_^2−^ ratio that simultaneous removal of COD, sulfate and production of sulfide effectively in SRBR, the continuous experiments were operated with HRT of 72 h for 180 days at various COD/sulfate ratios (0.5, 1, 1.5, 2, 2.5, 3, 4, 5, and 6). The period of each COD/sulfate ratio lasted for 20 days. After 20-day operation of COD/SO_4_^2−^ ratio of 0.5, other ratios (1, 1.5, 2, 2.5, 3, 4, 5, and 6) were respectively started up and having similar performance. During the optimization of the COD/SO_4_^2−^ ratio, sulfate concentration was maintained constant (1300 mg L^−1^) while the COD/sulfate ratios were increased in a stepwise manner from 0.5 to 6 by increasing the amount of sodium lactate (in COD). The sulfate removal efficiency was estimated by measuring the concentration of sulfide and sulfate in the effluent of SRBR with all ratios of COD/SO_4_^2−^ after reaching a dynamic equilibrium (after 7 days). The COD removal efficiency was only monitored at COD/SO_4_^2−^ ratios of 1.5, 2, 2.5 and 3 that strong sulfate-reducing activity was observed.

* Determination of suitable HRTs for sulfide removal and electricity generation in SOFC

For the effect of HRTs on the sulfide removal and the electricity generation capacity of SOFC, a feed COD/SO_4_^2−^ ratio of 2 and a sulfate concentration of 1300 mg L^−1^ were used. The effluent of the SRBR was supplied using a peristaltic pump into the SOFC system at various HRTs in the range of 12, 18, and 24 h. Sulfide concentrations in the influent and effluent of the anode chamber in SOFC were measured at 12 h intervals during 3 days of each HRT. The power density was examined every 2 h in SOFC.

* Performance of wastewater treatment system

Based on the selected COD/SO_4_^2−^ ratio of 2 and optimum HRT from the test in SRBR (72 h) and SOFC (24 h), respectively, continuous operation of the wastewater treatment system was carried out for 63 days. The SOFC was operated without the addition of external substrates or electron transport mediators during the experiment. After reaching a dynamic equilibrium (7 days), the influent and effluents from SRBR and SOFC were sampled for investigating removal efficiencies of sulfate, sulfide, and COD every day. The power density was monitored every 2 h to check for changes in the electricity generation efficiency during the operation.

### 4.3. Analytical Methods

Influent and effluent samples of SRBR and SOFC were collected over time for COD, sulfate, and sulfide measurement. Before each analysis, the samples were filtered through a 0.45 µm nitrocellulose membrane syringe filter. Sulfate was measured using the turbidimetric method based on the addition of barium chloride to form a colloidal suspension of barium sulfate at 420 nm [55]. Sulfide (H_2_S, HS^−^, and S^2−^) was measured at 480 nm according to Cord–Ruwish method based on CuS precipitation [56]. The organic substrate utilization was estimated by measuring the chemical oxygen demand (COD). The concentrations of COD were monitored in a concentrated sulfuric acid based on digestion with potassium dichromate for 2 h at 150 °C [55]. Power density (P, mW/m^2^) was counted according to P = IU/A. Where I (A) is current, U (mV) is voltage, and A (m^2^) is the surface area of the cathode.

The surfaces of the clean anode and the used anode were analyzed using scanning electron microscopy (SEM) (HITACHI, S—4800, Tokyo, Japan) equipped with an energy dispersive X-ray (EDX) detector (HORIBA, model 7593—H, Kyoto, Japan). For SEM examination, samples were first immersed in glutaraldehyde (2.5%, 60 min) and then washed with phosphate buffer (0.1 M, pH 7.0, 3 times). Finally, the samples were treated with critical point drying to dehydrate the biological tissues and coated with Pt.

## 5. Conclusions

In this study, the integrated treatment system consisting of an anaerobic SRBR and a SOFC has been successfully applied to treat organic wastewater containing high concentrations of sulfate/sulfide. Sulfide produced in the sulfate reduction process by SRB acts not only as an endogenous electron mediator but also as an electron donor to oxidize sulfide into non-toxic sulfur and recoverable by precipitation. High sulfate and COD removal were attained in SRBR at feed COD/SO_4_^2−^ ratios of 2 and sulfate concentration of 1300 mg L^−1^ under continuous operation at HRT of 72 h. The COD and sulfate removal efficiencies of SRBR were 94.8 ± 0.6 and 93 ± 1.3%, respectively, during the operation. The maximum sulfide removal efficiency achieved was as high as 93.7 ± 1.2% and power density reached 18.2 ± 1.6 mW/m^2^ with HRT of 24 h and initial sulfide of 316 ± 5.8 mg L^−1^. However, the sulfide removal efficiency and power density dropped gradually after 45 days of operation. At the end of the operation, sulfide removal efficiency and power density were 68% and 7.2 mW/m^2^, respectively. This might explain why the accumulation of sulfur on the anode may have decreased the electrical conductivity of the anode, thereby increasing the overpotential of the anode in the SOFC over time. The results presented in this study clearly revealed the feasibility of using an integrated treatment system to control the removal of pollutants from wastewater and electricity generation.

However, a major limitation of the method is the decrease in electrochemical activity over time due to the deposition of elemental sulfur. The precipitated sulfur forms a barrier towards the further oxidation of sulfides over long periods of time. Therefore, an efficient method to re-activate the electrode and recover sulfur from the electrode surface needs to be developed in the future.

## Data Availability

Not applicable.
